# Bioprocess optimization for pectinase production using *Aspergillus niger* in a submerged cultivation system

**DOI:** 10.1186/s12896-018-0481-7

**Published:** 2018-11-09

**Authors:** Hesham A. El Enshasy, Elsayed Ahmed Elsayed, Noorhamizah Suhaimi, Roslinda Abd Malek, Mona Esawy

**Affiliations:** 1 Institute of Bioproduct Development (IBD), Universiti Teknologi Malaysia (UTM), 81130 UTM, Skudai, Malaysia; 2 City of Scientific Research and Technology Application, New Burg Al Arab, Alexandria, Egypt; 3 Bioproducts Research Chair, Zoology Department, Faculty of Science, King Saud University, 11451 Riyadh, Kingdom of Saudi Arabia; 4 Chemistry of Natural and Microbial Products Department, National Research Centre, 12622 Dokki, Cairo, Egypt

**Keywords:** *Aspergillus Niger*, Pectinase, Medium optimization, Bioprocess optimization, Batch culture, Fed-batch culture

## Abstract

**Background:**

Pectinase enzymes present a high priced category of microbial enzymes with many potential applications in various food and oil industries and an estimated market share of $ 41.4 billion by 2020.

**Results:**

The production medium was first optimized using a statistical optimization approach to increase pectinase production. A maximal enzyme concentration of 76.35 U/mL (a 2.8-fold increase compared with the initial medium) was produced in a medium composed of (g/L): pectin, 32.22; (NH_4_)_2_SO_4_, 4.33; K_2_HPO_4_, 1.36; MgSO_4_.5H_2_O, 0.05; KCl, 0.05; and FeSO_4_.5H_2_O, 0.10. The cultivations were then carried out in a 16-L stirred tank bioreactor in both batch and fed-batch modes to improve enzyme production, which is an important step for bioprocess industrialization. Controlling the pH at 5.5 during cultivation yielded a pectinase production of 109.63 U/mL, which was about 10% higher than the uncontrolled pH culture. Furthermore, fed-batch cultivation using sucrose as a feeding substrate with a rate of 2 g/L/h increased the enzyme production up to 450 U/mL after 126 h.

**Conclusions:**

Statistical medium optimization improved volumetric pectinase productivity by about 2.8 folds. Scaling-up the production process in 16-L semi-industrial stirred tank bioreactor under controlled pH further enhanced pectinase production by about 4-folds. Finally, bioreactor fed-batch cultivation using constant carbon source feeding increased maximal volumetric enzyme production by about 16.5-folds from the initial starting conditions.

## Background

Pectic substances are a class of complex glycosidic polysaccharide compounds with a high molecular weight. They constitute the main components of the middle lamella and primary plant cell wall. Pectinase (EC 3.2.1.15) is an enzyme that hydrolyzes these biopolymers; it breaks down pectin in plant materials. This enzyme attacks and depolymerizes pectin through hydrolysis and esterification reactions. Pectinase splits polygalacturonic acid (pectate polymer) into monoglacturonic acid by opening the glycosidic linkages and breaking ester bonds between carboxyl and methyl groups [1]. This enzyme exists naturally in many plants; however, the industrial production of pectinase is carried out mainly using microbial systems. The food enzyme market was valued at about $1.4 billion in 2012, and it is expected to increase up to $ 41.4 billion by 2020, with a Compounded Annual Growth Rate (CAGR) of 6.7% [2]. In addition, pectinases make up almost 25% of the global food enzyme market [3]. A large pectinase market is required because of the wide range of applications in the food industry. This enzyme is widely used to degrade plant material in food production industries, as it accelerates fruit juice extraction [4, 5]. It is also used during juice and wine production, as it breaks down the fruit material, extracts flavors, and increases the clearness of the final product. Interestingly, pectinase reduces production costs in term of a higher yield, less equipment required, and less labor, especially during juice concentration [6, 7]. In addition to its extensive use in the juice and wine industries, it is now extended to textile, tea, coffee, and oil extraction and the treatment of pectin rich industrial waste water [8–10].

A large number of bacterial strains, mainly *Bacillus* spp., yeasts (*Yarawia lipolytica* and *Saccharomyces cerevisiae*), and many filamentous fungi, such as *Aspergillus niger*, *A. oryzae*, *A. awamori*, *A. sojae*, *Trichoderma viridiae*, *T. virens*, *Penicillium griseoroseum*, and *Phanerochaete chrysosporium*, are potential pectinase producers [3, 11–17]. Of these strains, *A. niger* has attracted the most attention as a pectinase bioreactory because of its long history in fermentation industries and its status as a Generally Regarded As Safe (GRAS) organism, according to the United States Food and Drug Administration [18]. Despite many reports on the potential uses of solid state fermentation (SSF) as an alternative cultivation system for pectinase production, submerged cultivation is by far the most favorable system for the large-scale production of pectinase, based on its ease in scalability, downstream processes, and optimization through different bio-processing approaches, such as fed-batch and continuous cultivation methods.

Fermentation medium accounts for a large portion of the production cost. Alternative substrates for pectinase production include cheap carbon sources including wheat bran, soybean meal, sugar cane molasses, and agro-industrial wastes, especially from citrus fruits such as sweet orange (*C. sinensis*) and lemon (*C. limon*) [3, 11]. In China and Southeast Asia, mandarin oranges (*C. reticulate*) are popular citrus fruits.

In this work, mandarin orange peel was used as a substrate for pectinase production at a semi-industrial scale, using a high yield-producing fungal strain, *A. niger* NRC1ami [19]. First, the medium was optimized for enzyme production using factorial and response surface designs. The Plackett-Burman Experimental Design (PBED) is based on the concept that every factor needs its own base level. Furthermore, it requires 4*n* experiments to investigate a maximum of 4*n*-1 factors at two levels [20]. The Box-Behnken (BB) response surface design produces second-order polynomial approximations to evaluate responses in certain regions. Both designs use statistical analysis which can improve both of upstream and downstream in many primary and secondary metabolites production processes [21–23]. This approach improves pectinase production quickly, with a significant reduction in medium costs [13]. The optimized medium was prepared in a 16-L stirred tank bioreactor using both batch and fed-batch cultivation strategies for pectinase production.

## Methods

### Extraction of pectin from mandarin peel

The pectin present in mandarin peel was extracted according to the method described by Huong and Luyen [24]. Briefly, 100 g of clean dried peels were mixed with 1 L of hot water, and then 500 mL 0.72% HCl were added. The mixture was incubated at 70 °C for 9 h, followed by separation of liquid phase. Finally, ethanol was added in an equivalent volume with gentle stirring to precipitate pectin, which was then centrifuged at 3000 rpm and dried overnight at 80 °C. The pectin concentration was determined gravimetrically using Ubbelohde viscometer 3 at 25 °C, where 1 g of dried pectin was dissolved in 100 mL 0.9% NaCl.

### Microorganism and inoculum preparation

*Aspergillus niger* NRC1ami was obtained from the National Research Center culture collection (NRC; Cairo, Egypt). This strain was isolated from citrus fruit and exhibits high extracellular pectinase production [19]. Once the culture was prepared in growing culture form, it was sub-cultured on potato dextrose agar (PDA) for 4 days at 30 °C. Spores were harvested in a 50% glycerol solution for preparation of the master cell bank, and the working cell bank was maintained in a cryogen vial at − 80 °C. Each experiment began by thawing and sub-culturing one vial on PDA. After 4 days of cultivation on an agar plate, *A. niger* spores were collected in saline solution.

### Medium for pectinase production and shake flask cultivation

The initial pectinase production medium was composed of (g/L): pectin, 30.0; (NH_4_)_2_SO_4_, 3.33; K_2_HPO_4_, 1.0; MgSO_4_.5H_2_O, 0.05; KCl, 0.05; and FeSO_4_.5H_2_O, 0.10. The pH was adjusted to 5.5 before sterilization.

### Shake flask and bioreactor cultivation

Shake flask cultivation was carried out in a 250-mL Erlenmeyer flasks with a 50 mL working volume, which were inoculated with 1 × 10^4^ spores/mL. The inoculated flasks were incubated in a temperature-controlled rotary shaker (Innova 4080, New Brunswick Scientific, Edison, NJ, USA) at 150 rpm and 30 °C. The propagated vegetative cells were used as inoculum for either shake-flask or bioreactor cultivations at a ratio of 5%.

A stainless steel, double-jacketed, 16-L stirred tank bioreactor was used (BioEngineering, Wald, Switzerland) with a working volume of 6 L. The stirrer was equipped with two, 6-bladed Rushton turbine impellers (d_i(impeller diameter)_ = 85 mm; d_t(tank diameter)_ = 214 mm, d_i_/d_t_ = 0.397). Sterilization was carried out at 121 °C for 30 min. The agitation was adjusted to 200 rpm throughout the cultivation, and aeration was performed using sterile air at a rate of 1 *v*/v/min. Foam was suppressed during cultivation using Struktul anti-foaming agent (Schill+Seilacher Grupper GmbH, Hamburg, Germany). The pH and dissolved oxygen were determined during the cultivation using pH and DO polarographic electrodes (Ingold, Mittler-Toledo, Greifensee, Switzerland). The pH-controlled culture was adjusted to 5.5 by connecting the pH controller to an acid/base feeding peristaltic pump containing 5 M HCl and 5 M NaOH solutions.

### Experimental design

The Plackett-Burman design was applied to determine the medium components affecting pectinase production. The four key medium components were pectin, (NH_4_)_2_SO_4_, K_2_HPO_4_ and MgSO_4_.5H_2_O; thus, these medium components were selected as variables. The low level (− 1) and high levels (+ 1) of individual nutrients are given in Table 1.

**Table 1:** Low and high levels of medium components affecting pectinase production by *A. niger* according to PBED

Factors	Low level (− 1)	High level (+ 1)
Pectin (g/L)	10.00	30.00
(NH_4_)_2_SO_4_ (g/L)	1.30	3.30
K_2_HPO_4_ (g/L)	0.50	1.00
MgSO_4_.5H_2_O (g/L)	0.05	0.50

The factors affecting the response of the Plackett-Burmann design were pectin, (NH_4_)_2_SO_4_ and K_2_HPO_4_. These three factors were further studied for the optimal range in the Box-Behnken design using Minitab 16 software (Minitab, Ltd., Coventry, UK). The low, center, and high levels are shown in Table 2.

**Table 2:** Low, medium and high levels of medium components affecting *A. niger* total enzyme activity and cell biomass in a BB design

Factors	Code	Low level (−1)	Middle (0)	High level (+ 1)
Pectin (g/L)	A	10.00	30.00	50.00
(NH_4_)_2_SO_4_ (g/L)	B	1.00	3.50	6.00
K_2_HPO_4_ (g/L)	C	0.50	1.25	2.00

The Box-Behnken design is based on the following second-order polynomial equation:

1 $$ Y={\beta}_0+\sum \limits_{i=1}^k{\beta}_i{x}_i+\sum \limits_{i=1}^k{\beta}_{ii}{x}_i^2+\sum \limits_{i<j}{\beta}_{ij}{x}_i{x}_j $$

where Y is the predicted pectinase activity (U/mL), *x*_*i*_ and *x*_*j*_ are the parameters (pectin, (NH_4_)_2_SO_4_, K_2_HPO_4_; g/L), *β*_0_ is the intercept term, and *β*_*i*_, *β*_*ii*_, and *β*_*ij*_ are the linear, squared, and interaction coefficients, respectively [25]. The predicted responses obtained from the Box-Behnken design were compared with the actual responses to estimate the accuracy of this methodology.

### Analyses

#### Biomass determination

During shake flask cultivation, samples were withdrawn intermittently for analysis. For the bioreactor cultivation, aliquots (20 mL) of the culture were taken through a sampling system. Samples were filtered using dry, pre-weighed Whatman filter paper. The supernatant was extracted to determine pectinase activity. The filtered biomass was washed twice using distilled water and dried in an oven at 100 °C until it reached a constant weight [26].

#### Enzyme assay

Pectinase determination was carried out using 1.0% (*w*/*v*) citrus pectin as the substrate; 0.3 mL of enzyme was added to 0.7 mL of substrate and mixed for 15 min at 40 °C. The liberated galacturonic acid concentration was determined using the method described in Esawy et al. [19]. One unit (U) of pectinase was defined as the amount of enzyme producing 1.0 μmol of galacturonic acid per min. Enzyme activity was calculated as:

$$ \mathrm{Activity},\mathrm{U}/\mathrm{mL}=\frac{\mathrm{galacturonic}\ \mathrm{acid}\ \mathrm{released},\upmu \mathrm{M}\ast \mathrm{Dilution}\ \mathrm{factor}}{\mathrm{Incubation}\ \mathrm{time},\min .} $$

## Results

### Plackett-Burman design screening of the main components affecting cell biomass and pectinase production by *A. niger*

Fractional factorial design was applied to find out the most significant medium components affecting pectinase production by *A. niger*. Table 1 shows different investigated medium components and their low (− 1) and high (+ 1) levels. The performed experiments (36 runs, Table 3) revealed that the highest pectinase activity of 87.73–88.85 U/mL was obtained from runs 2, 27 and 29, which were conducted using medium containing (g/L): pectin, 30.0; (NH_4_)_2_SO_4_, 3.3; K_2_HPO_4_, 0.50; MgSO_4_.5H_2_O, 0.50. Moreover, the coefficients of determination (R^2^) for both cell biomass and pectinase activity were 86.94 and 85.31%, respectively, indicating that this design had a good model fitting.

**Table 3:** PBED with four variables and the actual responses of total enzyme and cell biomass

Run	Pectin (g/L)	(NH_4_)_2_SO_4_ (g/L)	K_2_HPO_4_ (g/L)	MgSO_4_.5H_2_O (g/L)	Response pectinase (U/mL)	Response CDW (g/L)
1	30.00	1.30	0.50	0.05	65.52	2.20
2	30.00	3.30	0.50	0.50	87.73	2.08
3	10.00	1.30	1.00	0.50	23.00	1.70
4	30.00	3.30	1.00	0.05	52.32	1.80
5	30.00	3.30	0.50	0.50	63.22	1.88
6	30.00	3.30	1.00	0.05	8.94	1.75
7	30.00	1.30	0.50	0.05	51.88	2.33
8	10.00	3.30	1.00	0.50	38.87	1.75
9	10.00	3.30	0.50	0.05	52.99	1.00
10	30.00	1.30	0.50	0.05	58.70	2.45
11	30.00	1.30	1.00	0.05	32.92	1.89
12	30.00	1.30	1.00	0.05	34.90	2.25
13	10.00	3.30	1.00	0.05	33.79	1.00
14	30.00	1.30	1.00	0.50	33.95	3.10
15	10.00	1.30	1.00	0.50	23.00	2.00
16	30.00	1.30	1.00	0.50	34.74	3.05
17	10.00	3.30	1.00	0.05	38.47	1.18
18	30.00	3.30	0.50	0.50	71.71	1.65
19	30.00	1.30	1.00	0.50	33.16	2.65
20	30.00	3.30	0.50	0.50	54.74	2.10
21	10.00	1.30	0.50	0.50	39.66	1.65
22	10.00	1.30	0.50	0.50	42.92	1.75
23	10.00	1.30	0.50	0.05	36.25	1.40
24	10.00	3.30	0.50	0.05	54.89	0.90
25	10.00	3.30	1.00	0.50	38.71	1.65
26	10.00	3.30	1.00	0.50	38.79	1.70
27	30.00	3.30	0.50	0.50	88.85	1.85
28	10.00	3.30	1.00	0.05	43.15	1.25
29	30.00	3.30	0.50	0.50	88.29	2.30
30	10.00	3.30	0.50	0.05	53.94	0.95
31	10.00	1.30	1.00	0.50	23.00	1.85
32	30.00	3.30	1.00	0.05	45.69	1.70
33	10.00	1.30	0.50	0.50	46.17	1.70
34	10.00	1.30	0.50	0.05	42.04	1.30
35	10.00	1.30	0.50	0.05	30.46	1.35
36	30.00	1.30	1.00	0.05	30.94	2.07

**Fig. 1:**
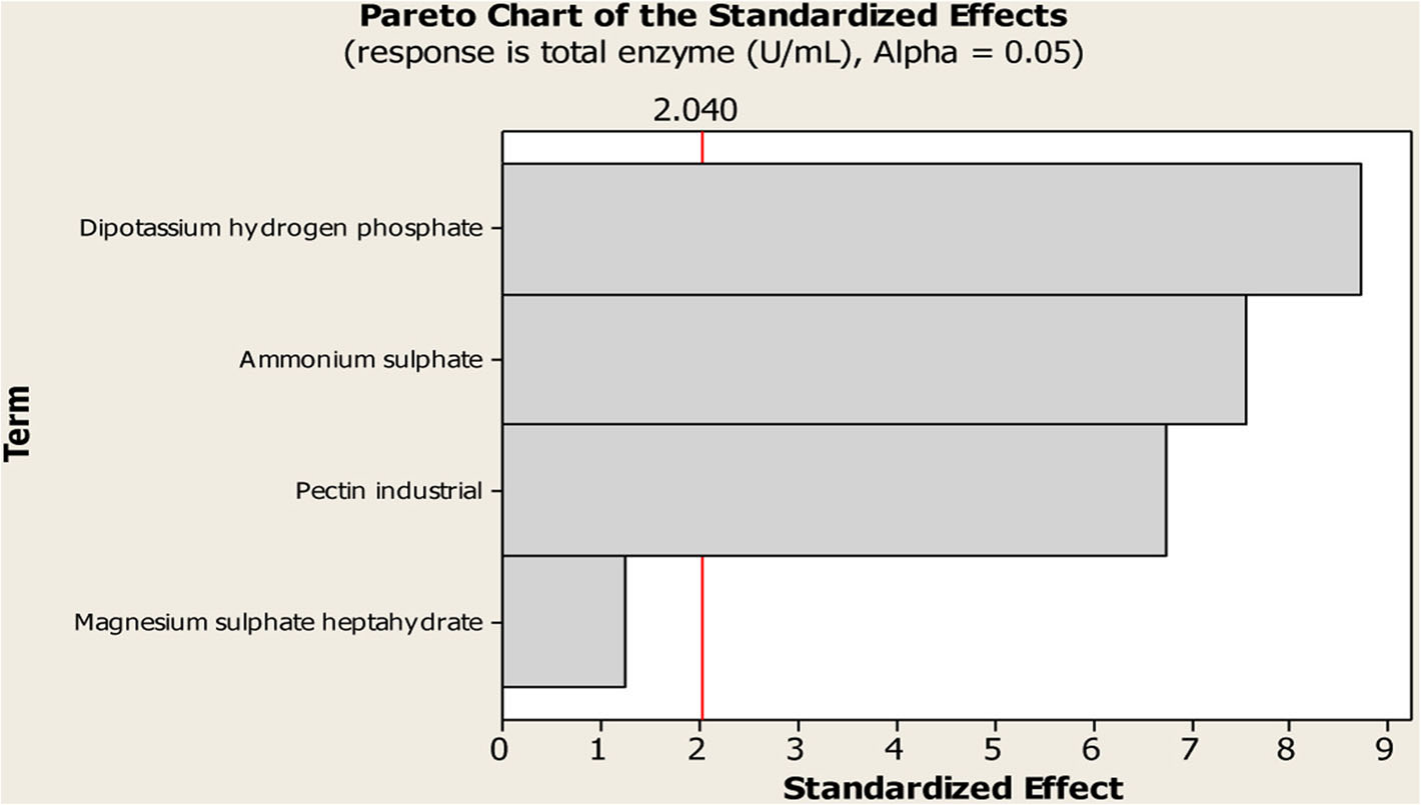
Pareto chart for the effect of the four factors on total enzyme production

However, based on the analysis of variance (ANOVA) for pectinase activity (Table 4, Fig. 1), it can be concluded that the first investigated medium components; i.e. pectin, (NH_4_)_2_SO_4_ and K_2_HPO_4_, were the most significant factors affecting pectinase production. This can be seen based on the obtained model *F*-value and the lower *p*-value (*p*- < 0.05). Accordingly, magnesium sulphate was excluded from the second step of optimization, since its effect on pectinase production was insignificant (*p*- = 0.23).

**Table 4:** Analysis of variance (ANOVA) for the pectinase activity model using BB design for the tested medium components

Source	DF	Sequential SS	Adjusted SS	Adjusted MS	*F* value	*p* value
Main effects	4	9095.90	9095.91	2273.98	45.01	0.00
Pectin	1	2305.20	2305.18	2305.18	45.62	0.00
(NH_4_)_2_SO_4_	1	2877.70	2877.72	2877.72	56.96	0.00
K_2_HPO_4_	1	3835.80	3835.82	3835.82	75.92	0.00
MgSO_4_.5H_2_O	1	77.2000	77.1800	77.18	1.530	0.23
Residual error	31	1566.30	1566.27	50.52		
Lack of fit	6	155.200	155.210	25.87	0.460	0.83
Pure error	25	1411.10	1411.06	56.44		
Corrected total	35	10,662.2				

*DF* Degree of freedom, *SS* Sum of squares, *MS* Mean sum of squares

### Statistical medium optimization using Box-Behnken design

The BB experimental design was further applied to optimize the concentrations of pectin, (NH_4_)_2_SO_4_ and K_2_HPO_4_ in the culture medium according to the three levels (low, − 1; middle, 0 and high + 1) studied and shown in Table 2. The obtained results (Table 5) clearly showed that highest pectinase activity was obtained upon using the middle levels of all tested three components (Runs 8, 10, 21, 22, 24 and 25), where the maximal pectinase activity obtained ranged from 84.88 to 101.06 U/mL.

**Table 5:** BB design of the experimental setup for response surface design with experimental values of total enzyme and cell biomass

Run	Pectin (g/L)	(NH_4_)_2_SO_4_ (g/L)	K_2_HPO_4_ (g/L)	Response pectinase (U/mL)	Response CDW (g/L)
1	50.00	3.50	0.50	30.00	1.75
2	50.00	6.00	1.25	39.00	2.00
3	50.00	3.50	0.50	32.00	2.00
4	10.00	6.00	1.25	71.55	1.30
5	30.00	6.00	2.00	83.29	1.55
6	50.00	1.00	1.25	29.00	1.95
7	10.00	1.00	1.25	50.61	1.20
8	30.00	3.50	1.25	101.06	1.95
9	50.00	3.50	2.00	26.00	2.10
10	30.00	3.50	1.25	97.41	1.65
11	10.00	1.00	1.25	50.45	1.30
12	10.00	6.00	1.25	70.28	1.10
13	50.00	6.00	1.25	40.00	2.75
14	30.00	6.00	0.50	66.95	1.30
15	10.00	3.50	0.50	62.99	1.15
16	10.00	3.50	2.00	61.56	1.15
17	50.00	3.50	2.00	23.00	1.90
18	30.00	6.00	2.00	79.96	1.50
19	10.00	3.50	0.50	60.92	1.05
20	30.00	1.00	2.00	85.04	1.40
21	30.00	3.50	1.25	98.05	1.60
22	30.00	3.50	1.25	96.62	1.65
23	30.00	1.00	0.50	59.18	1.85
24	30.00	3.50	1.25	86.62	1.65
25	30.00	3.50	1.25	84.88	1.70
26	50.00	1.00	1.25	25.00	1.65
27	30.00	1.00	0.50	64.57	1.75
28	30.00	1.00	2.00	81.86	1.65
29	30.00	6.00	0.50	63.62	1.55
30	10.00	3.50	2.00	61.87	1.25

The present results show that the applied model exhibited significant *p*-values for the effects of the investigated factors on pectinase production (Table 6). Thus, this model was considered highly significant. From the obtained ANOVA results for the regression model in Table 6, we can deduce the following polynomial:

**Table 6:** Estimated regression coefficients for the total enzyme production by *A. niger u*sing BB design

Term	Parameter estimate	SE Coeff.	T value	*p* value
Constant	- 30.90	2.85	33.00	0.00
A	4.89	1.75	- 8.81	0.00
B	12.28	1.75	2.47	0.02
C	56.8	1.75	2.23	0.04
A^2^	- 0.09	2.57	- 14.66	0.00
B^2^	- 1.51	2.57	- 3.67	0.00
C^2^	- 20.66	2.57	- 4.52	0.00

SE Coeff., Standard Error Coefficient

2 $$ Y\left( Pectinase,U/ mL\right)=-30.90+4.885A+12.28B+56.8C-0.09423{A}^2-1.508{B}^2-20.66{C}^2 $$

**Fig. 2:**
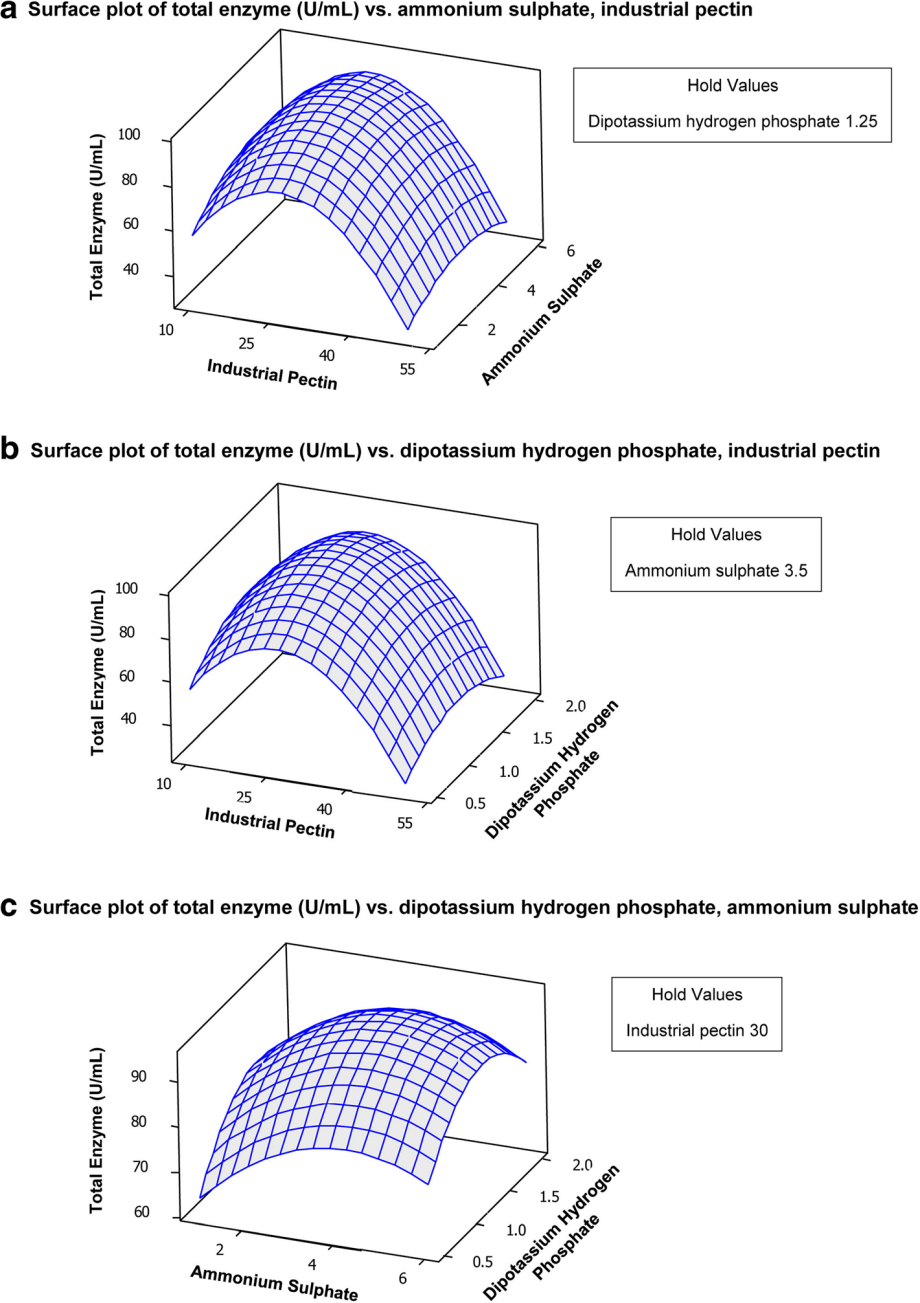
Contour plots of pecinase production by *A. niger* showing the interactions between pectin, (NH_4_)_2_SO_4_ and K_2_HPO_4_

Response surface plots (Fig. 2a-c) represent correlations between the experimental factors (pectin, (NH_4_)_2_SO_4_ and K_2_HPO_4_) and the response of pectinase production. Figure 2a shows the relative effects between (NH_4_)_2_SO_4_ concentration (1.0–6.0 g/L) and pectin concentration (10–50 g/L) while keeping K_2_HPO_4_ concentration constant at 1.25 g/L. Pectinase production increased with increasing pectin concentration to reach its maximal production at 32.22 g/L of pectin, while (NH_4_)_2_SO_4_ was less effective on pectinase production. Figure 2b shows the relative effects between K_2_HPO_4_ concentration (0.5–2.0 g/L) and pectin concentration (10–50 g/L), while keeping (NH_4_)_2_SO_4_ concentration constant at 3.5 g/L. Results showed that highest pectinase production will be obtained at the most optimum pectin concentration (32.22 g/L), while 1.36 g/L K_2_HPO_4_ was suitable for producing maximal pectinase production. On the other hand, the combined surface plot for (NH_4_)_2_SO_4_ and K_2_HPO_4_ (Fig. 2c), where pectin concentration was fixed at 30 g/L, shows that increasing both components gradually increased pectinase production up to its maximal, and that further increase resulted in decreased pectinase production. The final optimum concentration for both components were 1.36 and 4.33 g/L for K_2_HPO_4_, and (NH_4_)_2_SO_4_, respectively. Furthermore, from the model results compared to our experimental runs, it can be seen that the maximal obtained experimental pectinase production (90 U/mL) was in good agreement with the model predicted production (92.48 U/mL) with a desirability of 0.915. It can be generally seen, that the evaluated components are significant for pectinase production. Carbon and nitrogen sources are basic components of fermentation medium, where they are used for cellular growth and metabolic machinery, which is finally reflected on the production of pectinase.

### Growth kinetics of *A. niger* cultivated in un-optimized and optimized medium

**Fig. 3:**
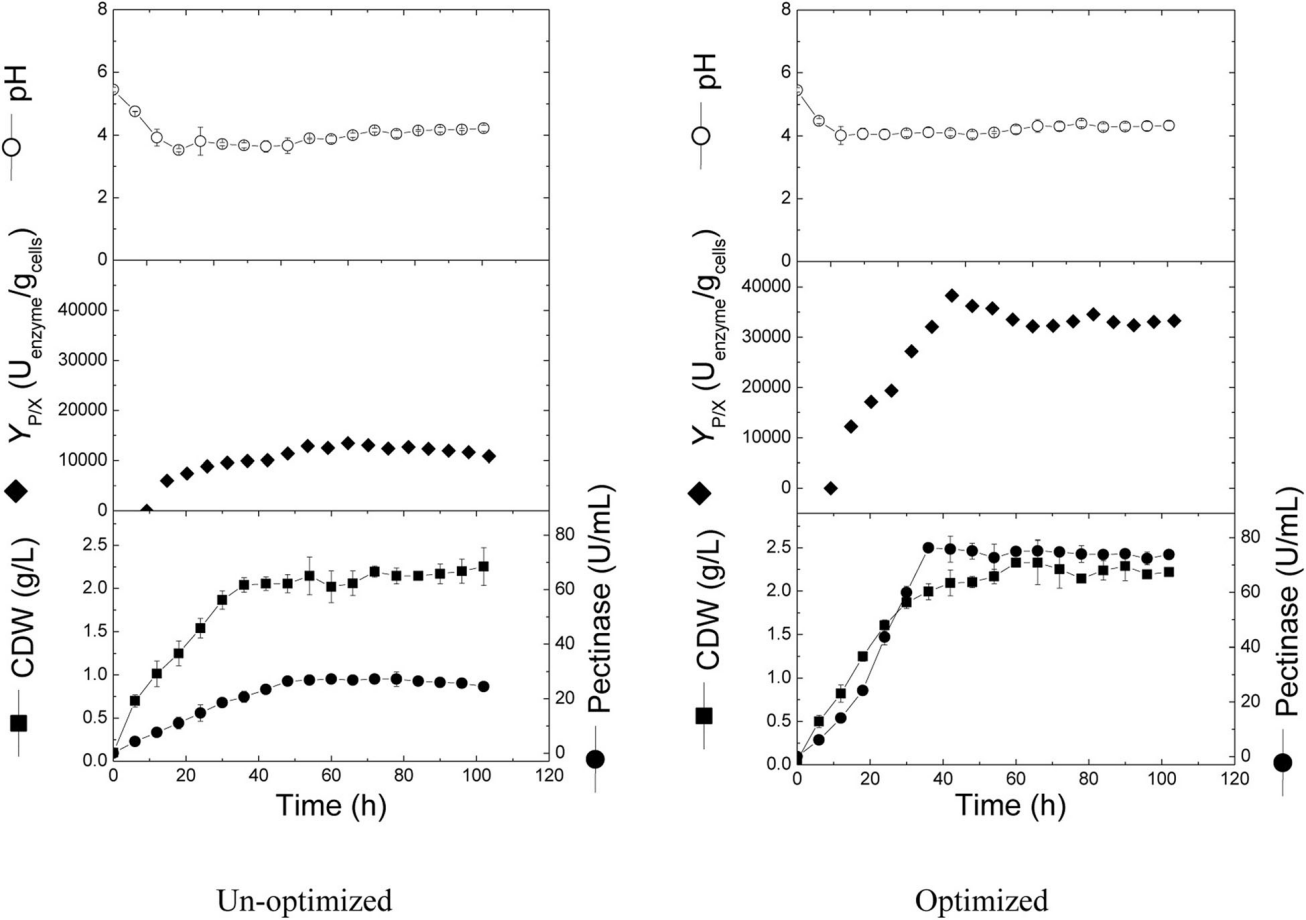
Cell growth, pectinase production, yield coefficient, and pH changes in the shake flask cultures using un-optimized and optimized media

This part of the work was designed to compare the kinetics of cell growth and pectinase production on both un-optimized and statistically optimized media in shake flasks (Fig. 3). Cells grew exponentially in both cultures for the first 36 h with a similar growth rate (0.054 g/L/h), after which, cell dry weight remained more or less constant till the end of fermentation. Maximal cell growth recorded comparable values by the end of both cultivations (2.26 and 2.29 g/L for un-optimized and optimized media, respectively). Moreover, during the early exponential phase (0–24 h), the pH dropped from 5.5 to 3.8 and 4.1 in un-optimized and optimized media, respectively, and remained approximately constant for the remaining cultivation time. On the other hand, pectinase production was significantly improved upon using optimized medium, where pectinase was produced at a production rate of about 2.12 U/mL/h and recorded a maximal production of 76.35 U/mL after 36 h. In contrast, in the un-optimized medium, the maximal pectinase achieved was 27.2 U/mL at 60 h, which was about 35.6% of the maximal pectinase produced in the optimized medium. Similarly, the production rate in the un-optimized was only 26.9% (0.57 U/mL/h) of the production rate in the optimized medium. Concerning production yield (*Y*_P/X_) results showed that the maximal yield obtained in optimized medium (38,270.7 U/g cells) was almost 3-folds of the maximal yield obtained in un-optimized medium (12,895.4 U/g cells).

### Batch cultivation in the bioreactor under controlled and uncontrolled pH

**Fig. 4:**
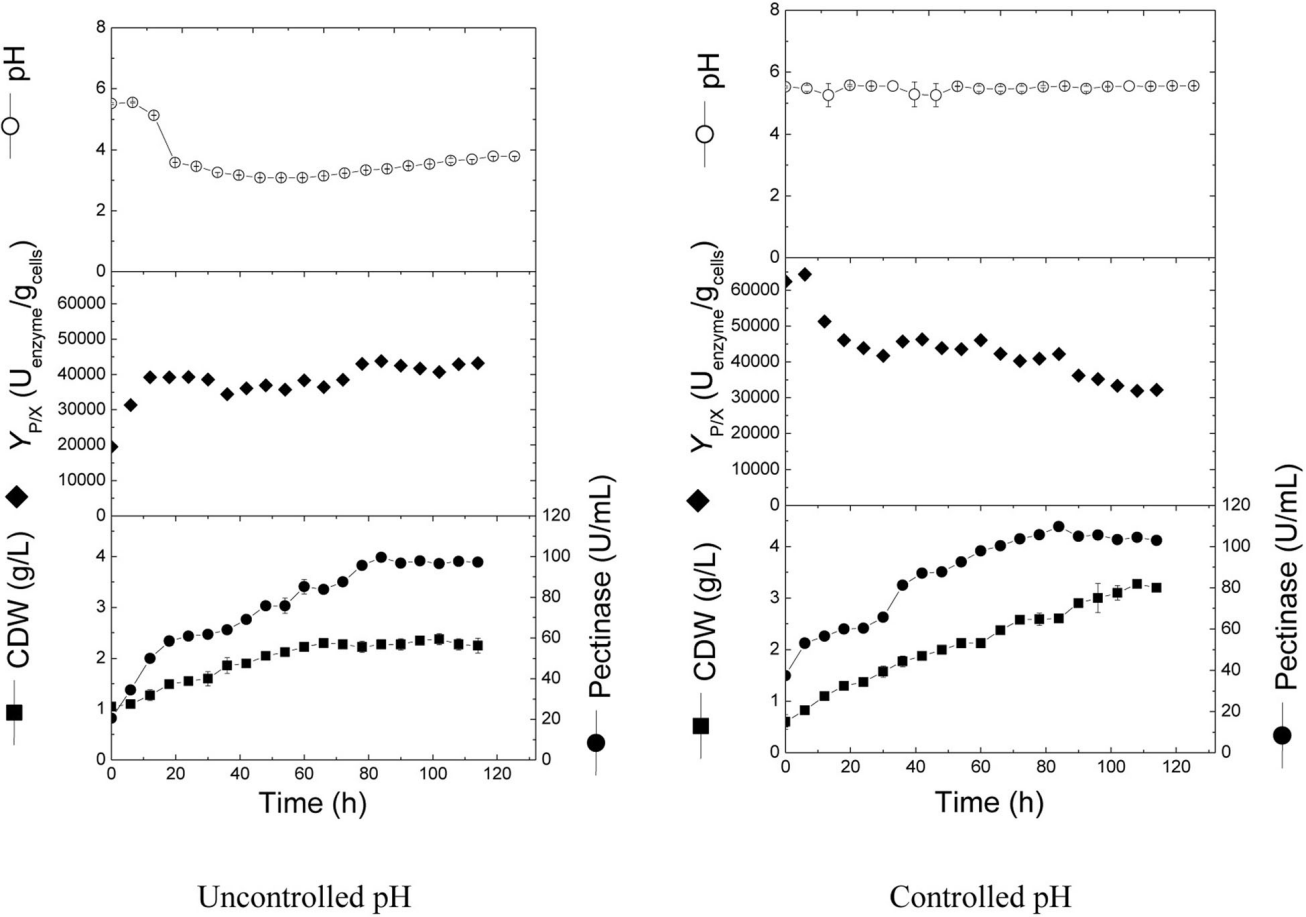
Cell growth, volumetric and specific pectinase production, and change in pH during batch cultivation of *A. niger* in a 16-L bioreactor under uncontrolled and controlled pH conditions

The optimized production conditions were transferred from shake-flask level to bioreactor level to gain insights about process performance in semi-industrial scale bioreactor. Cultivations were run in a 16-L stirred tank bioreactor at an aeration rate of 1 *v*/v/min, under uncontrolled pH. This cultivation was compared with cultivation conducted at controlled pH conditions at 5.5 by the continuous addition of H_2_SO_4_/NaOH using a computerized pH control system (Fig. 4). Results showed that the pH in the uncontrolled cultivation dropped from 5.5 to about 3.6 after 18 h, and remained more or less constant until the end of the cultivation. It can also be noticed that cells grew exponentially without a lag phase, in both cultivations. Maximal cell growth reached 2.38 and 3.28 g/L for uncontrolled and controlled pH, respectively. Additionally, the average cell growth rates were similar (0.02 and 0.028 g/L/h, for uncontrolled and controlled cultivations, respectively). Concerning pectinase production, results showed that the enzyme was exponentially produced until 84 h with production rates of 0.94 and 0.86 U/mL/h in pH-uncontrolled and -controlled cultivations, respectively. The maximal pectinase produced in pH-controlled cultivation (109.63 U/mL) was higher by about 10% from the maximal pectinase produced in pH-uncontrolled cultivation (99.55 U/mL). This increase in enzyme production can be attributed to the increase in cell biomass rather than the increase in cell productivity, since the maximal recorded production yields (*Y*_P/X_) were comparable in both cultivations (46,282.7 and 43,760.3 U/g cell for controlled and uncontrolled pH cultures, respectively). Additionally, both produced maximal pectinase concentrations were higher by about 30.4 (uncontrolled pH) and 43.6% (controlled pH) than the maximal pectinase produced in optimized shake flask cultivation (76.35 U/mL).

### Fed-batch cultivation in the bioreactor with constant sucrose feeding

**Fig. 5:**
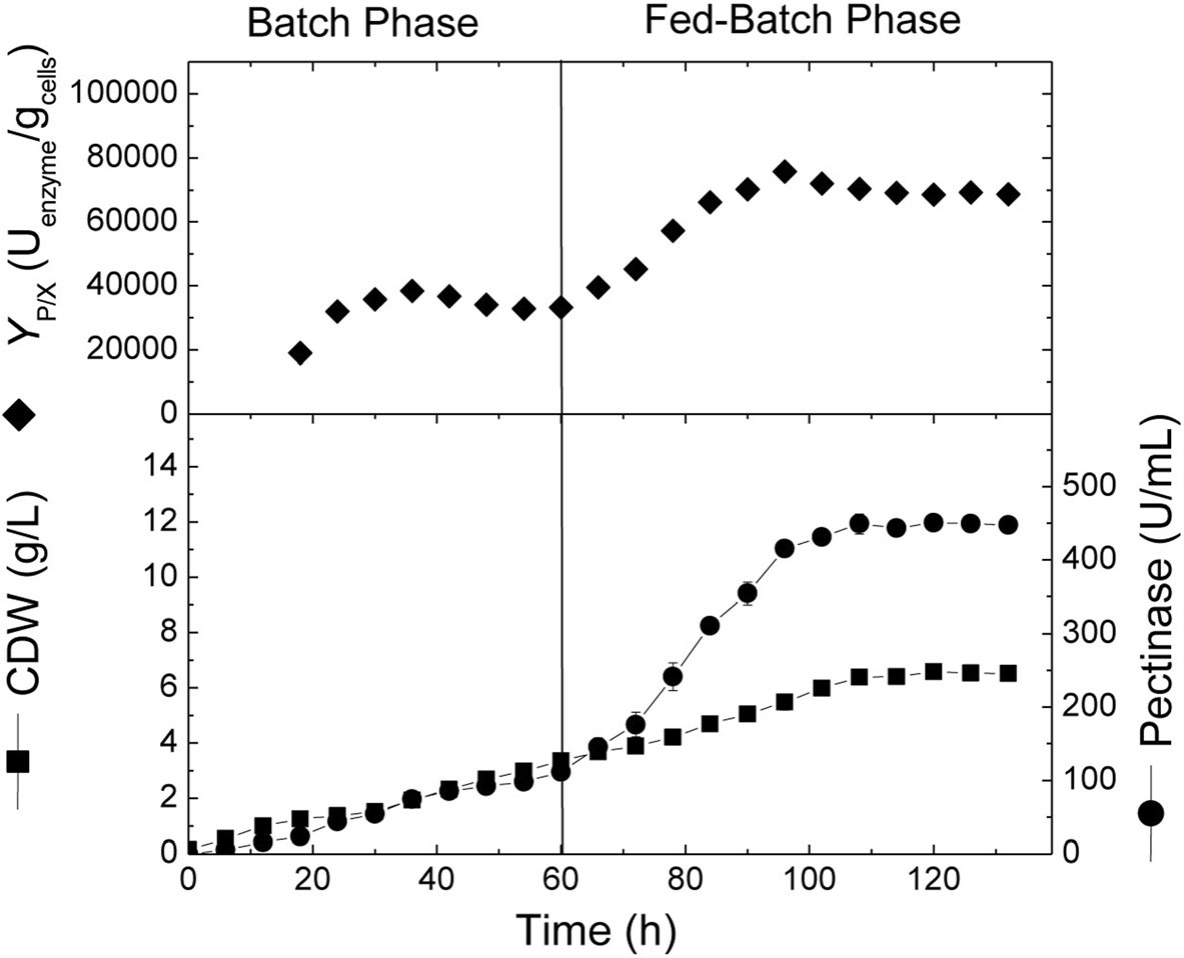
Cell growth, volumetric and specific pectinase production, and change in pH during fed-batch cultivation of *A. niger* in a 16-L bioreactor

Based on the previous data obtained from batch cultivations, fed-batch cultivation was conducted under controlled pH conditions in 16-L stirred tank bioreactor (Fig. 5). The cultivation was started as a normal batch mode for the first 60 h, where the cell growth and pectinase production kinetics were similar to the previous experiments, reaching maximal cell growth and pectinase production of 2.8 g/L and 120 U/mL, respectively. At 60 h, and before entering the stationary growth phase, sucrose was added at a constant feeding rate of 2.0 g/L/h. Accordingly, cells continued to grow exponentially with the same growth rate (0.028 g/L/h) and reached a maximal of 6.58 g/L after 120 h. Concomitantly, the volumetric pectinase production increased with an average production rate of 7.33 U/mL/h and reached a maximal of 450 U/mL at 108 h (about 4-folds higher than the corresponding batch culture). Afterwards, pectinase production remained approximately constant for the rest of cultivation. The highest production yield coefficient (*Y*_P/X_) obtained in the fed-batch cultivation recorded 75,762 U/g cells, which was almost 63.7% higher than the highest production yield obtained in pH-controlled batch cultivation (46,282.7 U/g cells).

Finally, Table 7 summarizes different steps applied for the development of the production bioprocess for pectinase enzyme. There were significant increases in both volumetric and specific enzyme production through medium optimization, bioreactor cultivation, and switching to fed-batch mode. The statistical media optimization enhanced the pectinase activity up to 76.35 U/mL, compared with only 26.85 U/mL in the un-optimized medium in shake flasks. Moreover, cells cultivated in the bioreactor under controlled pH conditions yielded the highest volumetric pectinase production of 109.63 U/mL. Pectinase production was maximized using sucrose feeding. The highest enzyme production was 450 U/mL after 108 h, which represented about 16-folds increase in volumetric production, compared with the initial un-optimized culture medium and conditions.

**Table 7:** Kinetic Parameters for *A. niger* cell growth and pectinase production under different cultivation conditions

Parameters	Shake Flask		Bioreactor		
	Un-optimized Medium	Optimized Medium	Batch		Fed-batch
			Controlled pH	Uncontrolled pH	
X_max_ (g/L)	2.26	2.29	3.28	2.38	6.58
μ (h^−1^)	0.01	0.01	0.01	0.01	n.d.
Pec_max_ (U/mL)	27.2 (60 h)	76.35 (36 h)	109.63 (84 h)	99.55 (84 h)	450 (108 h)
Q_Pec_ (U/mL/h)	0.57	2.12	0.86	0.94	7.33
*Y*_Pec/X_ (U/g)	12,517 (48 h)	38,270 (36 h)	46,283 (42 h)	43,760 (84 h)	75,762 (94 h)

*n.d.* Not determined

X_max_: maximal cell dry weight, μ: specific growth rate; Pec_max_: maximal pectinase production; Q_Pec_: pectinase production rate; *Y*_Pec/X_: yield of pectinase on biomass

## Discussion

Pectinase enzymes constitute a major sector in the enzyme food market [3, 5, 7]. Accordingly, efforts have been carried out to seek novel compositions of production medium components as well as developed methods for semi-industrial production. In our present study, we investigated the fractional factorial design approach for initial optimization of medium components. Our results showed that pectin, (NH_4_)_2_SO_4_ and K_2_HPO_4_, were the most significant factors affecting pectinase production. Furthermore, determination coefficients (R^2^) for cell biomass and pectinase activity were 86.94 and 85.31%, respectively, representing a good model fitting. These results are in good agreement with those previously reported for statistical medium optimization for pectinase production using either fungal or bacterial microorganisms [5, 27, 28]. Authors concluded that MgSO_4_ is not significantly affecting pectinase production during their statistical optimization experiments. Although Mg^+ 2^ acts generally as an inducer or co-factor for enzyme production, however, few reports have suggested that magnesium sulphate can inhibit glucose 6-phosphate dehydrogenase, G6PD [29, 30]. G6PD is a key enzyme in the fungal pentose phosphate pathway responsible for the production of NADPH and is directly correlated with biomass and hence enzyme production [31, 32].

Secondly, Box-Behnken design was performed in order to further optimize the concentrations of the obtained most significant components; pectin, (NH_4_)_2_SO_4_ and K_2_HPO_4_. We found that highest pectinase activity was obtained using the middle levels of all tested three components (Table 5: Runs 8, 10, 21, 22, 24 and 25). Our obtained results can be correlated with those obtained by Ghazala et al. [10] who investigated statistical optimization of medium components for the production of pectinase by *Bacillus mojavensis* using carrot peel powder as a substrate. Their results also found that the middle level of carrot peel powder (0) was the best level for the production, and that pectinase levels decreased significantly when the higher levels were used (+ 1). The model for pectinase activity was used to calculate the coefficient of determination (R^2^), which can be defined as the ratio of the expected deviation to the overall deviation. Our results showed that R^2^ has a value of 93.69% for pectinase response, which indicates a good fitting of the model and that the model can explain 93.69% of the deviation from the expected values, and that only 6.31% of the deviation cannot be explained by the proposed model. Moreover, as the value of R^2^ comes closer to 1.0, this means that the model correlates well [5]. Furthermore, the obtained results are in good accordance with those reported by Tari et al. [13], where they stated that the *p*-value reflects the relationship between the variables/factors and the response variable, i.e. *p*-value lower than 0.05 indicates that the applied model is significant.

The comparison of cultivation performance between un-optimized and optimized medium composition in terms of cell growth and pectinase production was carried out. Although, results showed similarities corresponding to cellular growth patterns in both conditions, however, pectinase production significantly increased upon medium optimization in terms of production rate and production yields. The obtained results are generally in good agreement with those reported in the literature for pectinase production. Pectinase literature shows that the maximal enzyme production is usually achieved at early growth stages (24–30 h), which are in good agreement with our results [33]. Statistical approaches have been applied for the optimization of medium components affecting pectinase production. Tari et al. produced pectinase efficiently and cost effectively using statistical optimization methods [13]. Furthermore, Kuvvet et al. applied statistical designing approaches to optimized cultivation medium containing apple pomace for the production of pectinase using *Bacillus* spp. [17]. They were able to obtain a 2-fold increase in pectinase production using Box-Behnken response surface methodology.

*A. niger* is an aerobic microorganism and thus needs a continuous supply of oxygen during cultivation for growth and efficient metabolite production [34]. Therefore, cultivations were run in a 16-L stirred tank semi-industrial bioreactor under both uncontrolled and controlled pH conditions. Results showed that bioreactor cultivations significantly improved cellular growth as well as pectinase production parameters in comparison to shake flask cultivations. Additionally, pH-controlled conditions favored maximal cell growth and pectinase production over pH-uncontrolled conditions. The aforementioned results clearly demonstrate the superiority of bioreactor cultivations over shake flask ones. This can be explained due to the improved oxygen availability and agitation conditions present in bioreactor cultivations [22, 35]. Moreover, the production of industrially important enzymes, i.e. pectinase, amylase and invertase, has been greatly improved upon scaling up the cultivation vessels from shake flask level to bioreactor level [32, 36]. Furthermore, the obtained results showed that the pH control led to about 10 and 27% increase in pectinase production and cell growth, respectively. This can be referred to the fact that controlling the pH of the cultivation provides the growing cells with much more stable cultivation environment which is reflected in the increased cell growth and production. The stability of the cultivation environment was found to be correlated with the intrinsic pathways for nutrient assimilation, cell growth and enzyme productivity [37]. Additionally, the effect of pH change on enzymes activity has been correlated with enzyme denaturation and destabilization of conformational structures [31].

On the other hand, both bioreactor cultivations showed lower rates of growth and pectinase production than in the case of shake flask cultivations. This decrease can be attributed to the nature of growing fungal hyphae, which under bioreactor conditions tend to have condensed morphology and thus affecting the viscosity of the fermentation broth. Consequently, the cultivation will suffer from decreased oxygen transfer between the condensed hyphae and the viscous broth leading to decreased growth and production rates [38, 39]. Moreover, Friedrich et al. reported that oxygen transfer and viscosity problems in bioreactors greatly affected pectinase production by *A. niger* and they were able to overcome such problems by increasing the agitation from 300 to 600 rpm and the aeration rate from 0.5 to 1.2 *v*/v/min once they reached their maximal growth rate [40]. Accordingly, they were able to obtain 2-fold increase in pectinase production. In our work, bioreactor cultivations were run at 1 v/v/min, in order to avoid increasing shear stress and consequently decreasing pectinase productivity. Furthermore, the decrease in growth and production rates in bioreactor cultivations can be explained due to the induction-repression or activation-inhibition mechanisms of pectinase production by *A. niger* [4, 41, 42]. The authors proposed that pectinase production is inhibited by catabolite repression through galacturonic acid units produced by the action of the enzyme on pectin, and that the galacturonic acid may be indirectly associated with the activation/inhibition mechanisms of pectinase production depending on its concentration in the medium.

Batch cultivation is generally terminated earlier due to exhaustion of important nutrient components from the cultivation medium, which greatly affects the overall productivity of the cultivation process. Therefore, fed-batch mode of cultivation was developed to overcome problems encountered in batch cultivations [36, 43]. Our obtained results showed a great improvement in pectinase production process upon feeding sucrose, where the volumetric production increased by about 4 folds. These results are in good accordance with those reported earlier in the literature concerning pectinase production. Tuttobello and Mill used pectin for the production of pectinase with *A. niger* [44]. They found that incorporating sucrose at a 4% concentration to the pectin medium greatly enhanced pectinase production, and that cells were able to utilize about 85% of the pectin contents in the medium. They also found that other carbohydrates tested did not show similar promoting effects. This can be supported with the work of Solís-Pereira et al. [4] who investigated the effects of different carbon sources on pectinase production by *A. niger*. They found that feeding sucrose to pectin containing medium greatly improved pectinase production due to its inductive effect. Moreover, Phutela et al. used wheat bran medium for the production of pectinase by *A. fumigatus* [45]. They found that supplementing their medium with sucrose resulted in about 37.8% increase in pectinase production than the control medium without sucrose addition.

## Conclusions

In the present work, a new strain, *Aspergillus niger* NRC1ami, isolated from citrus fruit, showed high pectinase production using a statistically optimized medium. The medium variables affecting enzyme production were pectin, (NH_4_)_2_SO_4_ and K_2_HPO_4_, while MgSO_4_ was found to be insignificant. The optimum medium composition statistically optimized (g/L) was: pectin, 32.22; (NH_4_)_2_SO_4_, 4.33; K_2_HPO_4_, 1.36; MgSO_4_.5H_2_O, 0.05; KCl, 0.05; and FeSO_4_.5H_2_O, 0.10. Medium optimization enhanced pectinase production by about 2.8 folds (from 27.2 to 76.35 U/mL). The enzyme yield was further improved by about 4-folds upon transferring the process from shake flasks to bioreactor under controlled pH. Further improvement was achieved by fed-batch cultivation with constant carbon source feeding on a semi-industrial scale in 16-L stirred tank bioreactor. Maximal volumetric enzyme production increased by about 16.5-folds from the initial starting conditions. Finally, the high yield obtained in the semi-industrial scale bioreactor supports the scaling-up and industrialization of this process.

## Abbreviations

ANOVA: Analysis of variance
BB: The Box-Behnken
CDW: Cell Dry Weight
PBED: Plackett-Burman Experimental Design
